# Prognostic Impact of Monocyte to Lymphocyte Ratio in Clinical Outcome of Patients with Hepatocellular Carcinoma: A Systematic Review and Meta-analysis

**DOI:** 10.31661/gmj.v9i0.1948

**Published:** 2020-12-25

**Authors:** Masoud Nouri-Vaskeh, Mohammad Mirza-Aghazadeh-Attari, Fariba Pashazadeh, Saber Azami-Aghdash, Hadi Alizadeh, Parnia Pouya, Monireh Halimi, Golamreza Jadideslam, Mohammad Zarei

**Affiliations:** ^1^Immunology Research Center, Tabriz University of Medical Sciences, Tabriz, Iran; ^2^Network of Immunity in Infection, Malignancy and Autoimmunity, Universal Scientific Education and Research Network, Tehran, Iran; ^3^Aging Research Institute, Tabriz University of Medical Sciences, Tabriz, Iran; ^4^Iranian EBM Centre: A Joanna Briggs Institute Affiliated Group; ^5^Research Center for Evidence-Based Medicine, Health Management and Safety Promotion Research Institute, Tabriz University of Medical Sciences, Tabriz, Iran; ^6^Tabriz Health Services Management Research Center, Health Management and Safety Promotion Research Institute, Tabriz University of Medical Sciences, Tabriz, Iran; ^7^Faculty of Medicine, Tabriz University of Medical Sciences, Tabriz, Iran; ^8^Department of Pathology, Faculty of Medicine, Tabriz University of Medical Sciences, Tabriz, Iran; ^9^Department of Molecular Medicine, Faculty of Advanced Medical Sciences, Tabriz university of Medical Sciences, Tabriz, Iran; ^10^Department of Pathology and Laboratory Medicine, Perelman School of Medicine, University of Pennsylvania, Philadelphia, PA, USA; ^11^Center for Mitochondrial and Epigenomic Medicine, Children’s Hospital of Philadelphia, Philadelphia, PA, USA

**Keywords:** Hepatocellular Carcinoma, Liver Neoplasm, Lymphocyte to Monocyte Ratio, Survival, Disease-Free Survival

## Abstract

**Background:**

Lymphocyte to monocyte ratio (LMR) is a surrogate marker of systemic inflammation which is shown to be related to the patient’s survival in multiple malignancies. An important implication of this marker potentially is neoplasms in which there is no correlation between prognosis and histopathological staging and also has no reliable chemical markers associated with prognosis. Herein, this meta-analysis aimed to investigate the prognostic role of LMR in patients with hepatocellular carcinoma (HCC).

**Materials and Methods:**

In the current systemic review and meta-analysis, we conducted a systemic search of databases and indexing sources, including PubMed, EMBASE, Cochrane, Scopus, and ProQuest up to May 2019 toinclude studies on the prognostic significance of LMR on patients with HCC. Overall survival (OS), disease-free survival (DFS) and recurrence-free survival (RFS) values were extracted from the studies and analyzed. The pooled hazard ratio with a 95% confidence interval was explored to identify the prognostic value of the LMR in the survival of the patients with HCC.

**Results:**

A total of 12 studies with a total sample size of 3750 cases were included. There was significant heterogeneity among the studies; therefore, subgroup analysis was also performed. Overall analysis regarding OS showed an insignificant relationship between LMR and patient’s prognosis, dividing to subgroups based on LMR cut-offs did not yield any significant result, subgroup analysis for RFS founded statistically significant results and LMR was significantly related to DFS.

**Conclusion:**

High LMR was associated with increased DFS and RFS, in return this association was not observed for OS.

## Introduction

Hepatocellular carcinoma (HCC) is one of the major causes of cancer-related death worldwide, with an un-proportionate incidence in developing countries, because of increased exposure to risk factors such as chronic viral hepatitis, aflatoxin generated in canned foods, and an increasing rate of smoking and obesity [1]. Much attention has been put into the association of these risk factors and cancer progression in the liver, and although a wide range of possible step-wise models of activation of pathologic mechanisms has been suggested, the inflammatory pathways are highly considered to be involved [2]. Inflammation is a major contributor to malignant transformation; it enables the creation of reactive oxygen species (ROS) that promotes further DNA damage. It is also a trigger for activation of cellular signaling pathways which promotes cellular proliferation and limit the extent of apoptosis. The NF-KB signaling pathway, the master regulator of inflammatory signaling, is a regulator of DNA-damage response end-points determining how cells react to external stressful stimuliwith potent pro-carcinogenic effects [3,4]. Another major influence of inflammation on cancer progression is exerted via inflammation mediators, mainly via cellular components of the immune system. It is well documented that the cells in the innate and adaptive immune systems are important agents to limit carcinogenesis by inducing apoptosis and autophagy in cancer cells; however, these cells are also able to secret DNA-damaging agents leading to carcinogenesis [5]. Although much debate exists about the overall effects of immune cells in cancer progression experimental studies on humans and disease models have found that being in a chronic state of immune stimulation is associated with poor prognosis [6]. Moreover, according to Zhao * et al.* study, low pretreatment lymphocyte count may represent an unfavorable prognostic factor for clinical outcomes in patients with solid tumors [7]. Recent meta-analysis were evaluated the prognostic role of blood inflammatory markers such as lymphocyte to monocyte ration in the HCC [8] and further meta-analyses in these inflammatory markers are necessary.

Lymphocyte to monocyte ratio (LMR) is a marker of immune activation, which has shown linked to the overstimulation of the immune system. Increased LMR is associated with an unfavorable prognosis [9], but how exactly LMR affects the tumor environment and the relationship between tumor stage, LMR and prognosis are still unknown, and more importantly, the results from different studies are in-consistent [10]. In addition, a growing number of articles are assessing the prognostic significance of LMR, making a systemic review, and meta-analysis of further beneficence. Moreover, few studies have explored the predictive ability of LMR in patients with HCC. Herein, we conduct a systematic review and meta-analysis to clarify the association between LMR and patients prognosis.

## Materials and Methods

 This study was conducted according to the Preferred Reporting Items for Systematic review and Meta-Analysis (PRISMA) for reporting in systematic review and meta-analysis and was registered at the PROSPERO (approval ID: CRD42019128454).

###  Search Strategies

 We searched PubMed, EMBASE, Cochrane, Scopus, and ProQuest databases up to May 2019 using the following keywords: (“liver tumor” OR “liver cancer” OR “liver neoplasms” OR “liver cell carcinoma” OR “hepatocellular carcinoma” OR “HCC”) AND (“LMR” OR “lymphocyte to monocyte ratio” OR “lymphocyte monocyte ratio” OR “lymphocyte-to-monocyte ratio” OR “lymphocyte-monocyte ratio”) AND (“survival” OR “prognostic” OR “prognosis” OR “outcome” OR “recurrence” OR “clinical outcome”). Reference lists of all articles found in the search were also screened for additional studies. References from relevant articles were also included and gathered manually. Moreover, handle search performed for possible more relevant studies.

###  Inclusion Criteria 

 Inclusion criteria were as follow: 1) All relevant observational and clinical prospective and retrospective studies; 2) The definite diagnosis of HCC by biopsies; 3) Studies assessing the association between LMR and overall survival (OS), diseases free survival (DFS), and relapse-free survival (RFS); 4) Reporting the cut-off value of LMR; 5) Providing enough information to calculate Hazard ratio (HR) with a 95% confidence interval [CI]; 6) Articles published in English

###  Exclusion Criteria

 The following exclusion criteria were also considered: 1) Experimental studies on animals; 2) Letters, editorials, expert opinions, reviews, and case reports; 3) Patients with other types of primary neoplasia; 4) Studies providing insufficient data.

###  Data Extraction

 Data extraction was done by two independent reviewers (HA and PP). Any disagreement between the two reviewers regarding the inclusion of the studies was resolved by a third reviewer (MN).

###  Quality Assessment

The Newcastle-Ottawa scale (NOS) was used to assess the reporting quality of the studies. This scale comprises of 9 distinct items in which each item is scored as one point. The maximum points available for each article is 9 and studies with scores higher or equal to 7 are considered to be high-quality studies [11]. In this review, all studies with a score of more than or equal to 6 were included.

###  Statistical Analysis 

Pooled analyses were performed using Comprehensive M/a-Analysis v. 2.0 (CMA, Biostat Inc., Englewood, NJ, USA). The prognosis outcomes were explored using the HR with corresponding 95% confidence interval. The prognosis outcomes mainly contained the OS, DFS or RFS. The heterogeneity was assessed across all studies by Cochran’s Q test and Higgins I^2^. The heterogeneity was considered significant when P<0.05 and/or I^2^≥50%, the random-effects model was used; if not, the fixed-effect model was applied. In addition, the funnel plot was conducted to evaluate publication bias. P≤0.05 was considered statistically significant

## Results

**Figure 1:**
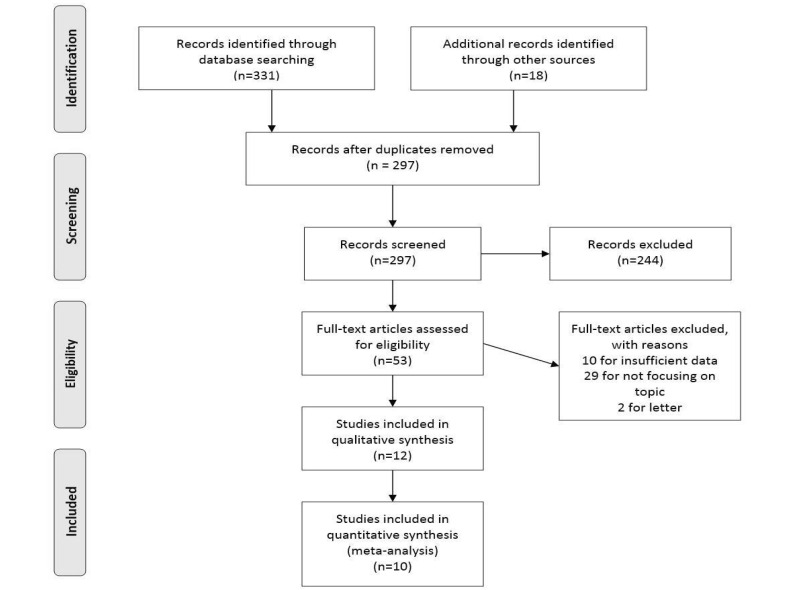
PRISMA diagram of the study

 The electronic search which was described above resulted in the identification of 331 articles which were eligible for inclusion in this study. PRISMA diagram of the study is shown in Figure-1 In the manual search, 18 new studies, which were not included in the electronic search, were identified. After the exclusion of duplicate articles, 297 articles remained. The abstract of all these articles was scanned and 244 articles were excluded. The remaining 53 articles underwent a full-text evaluation, of which 41 were excluded. The remaining 12 studies were subjected to a final review, and 10 were included in the final meta-analysis (abstracts were not included in quantitative synthesis). Of the 12 included studies, 11 reported OS, 5 reported RFS, 2 reported DFS and one study had reported time to recurrence (TTR). The combined sample size of the studies was 4253 patients, and the sample size of the studies being analyzed was 3750 patients. The quality of the studies was reported based on the NOS scale. All studies scored 6 or above and thus all of them were of sufficient quality. Table-1 summarizes the main findings of the 12 studies included in the systemic review and the 10 original articles analyzed.

###  1. Meta-analysis

####  1. 1.OS

**Figure 2:**
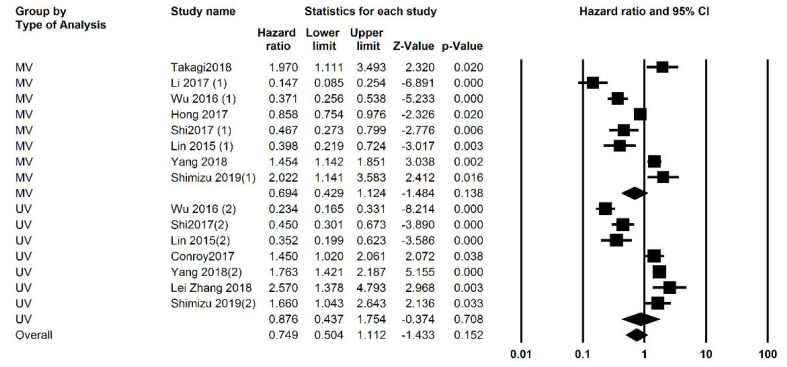
Analysis of the predictive value of lymphocyte to monocyte ratio in overall survival of hepatocellular carcinoma

**Figure 3:**
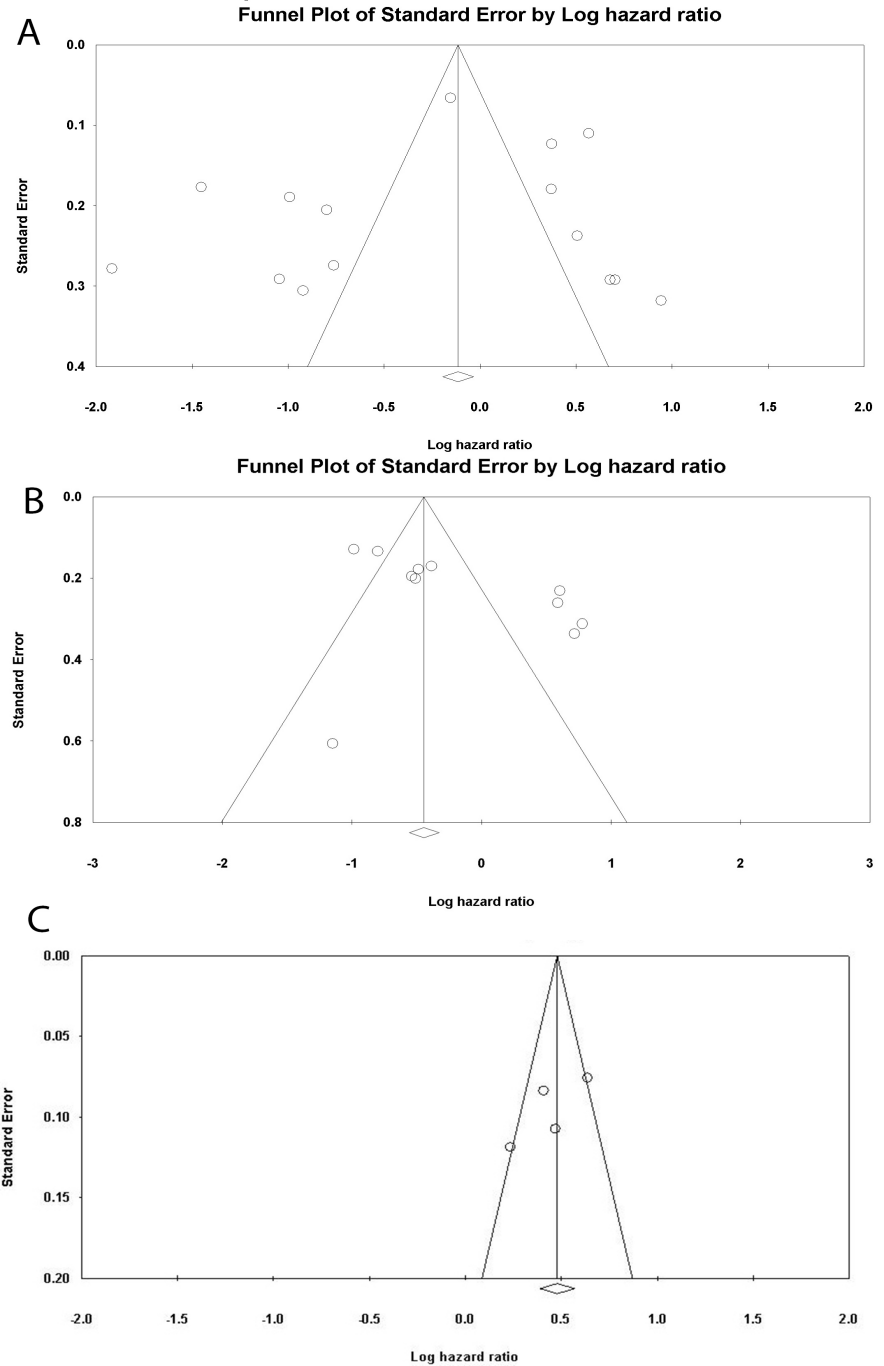
Funnel plot of the predictive value of lymphocyte to monocyte ratio (LMR) in overall survival of hepatocellular carcinoma, **B.** Funnel plot of the predictive value of LMR in recurrence-free survival, **C.** Funnel plot of the predictive value of LMR in disease-free survival

**Supplementary Figure 1:**
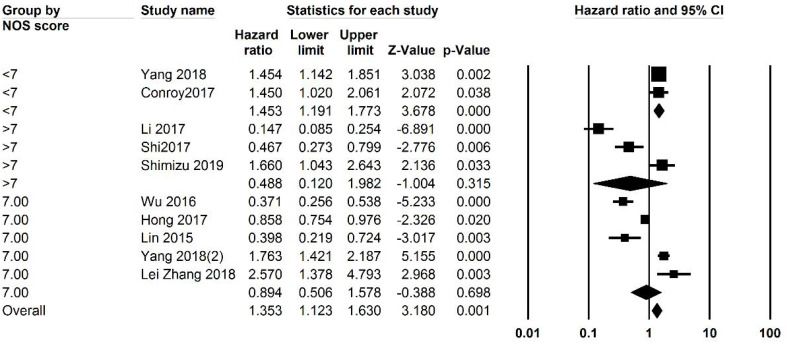
Subgroup of the predictive value of lymphocyte to monocyte ratio in overall survival based on Newcastle-Ottawa scale of studies being included

**Supplementary Figure 2:**
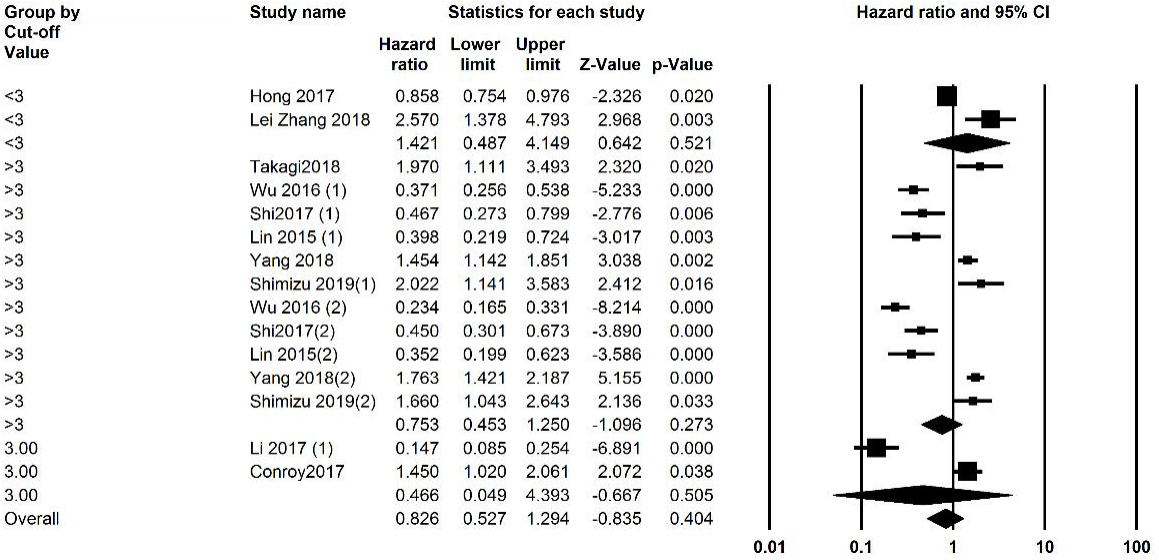
Subgroup analysis of the predictive value of lymphocyte to monocyte ratio (LMR) in overall survival based on the determined cut-off value for LMR

 Studies were analyzed for heterogeneity, and I2 was equal to 94.388, showing a high level of heterogeneity. Because of this subgroup analysis was also performed. Both univariate and multivariate analysis were included and analyzed separately, and then an overall analysis was performed. Overall 3059 patients were analyzed and the overall HR was 0.749 (95% CI: 0.504 -1.112, P=0.152, Figure-2). Subgroup analysis was performed and revealed that in studies with a NOS score of less than 7 (P=0.001). Analysis based on the cut-off points did not generate a significant association. Subgroup analysis was also performed based on cut-off values, and studies were divided into those with a cut-off value of less than 3 and more than 3. P was equal to 0.505 for both groups. The funnel plot of the OS analysis is included in Figure-3A, S1, and S2.

####  1. 2.Recurrence -free Survival 

**Figure 4:**
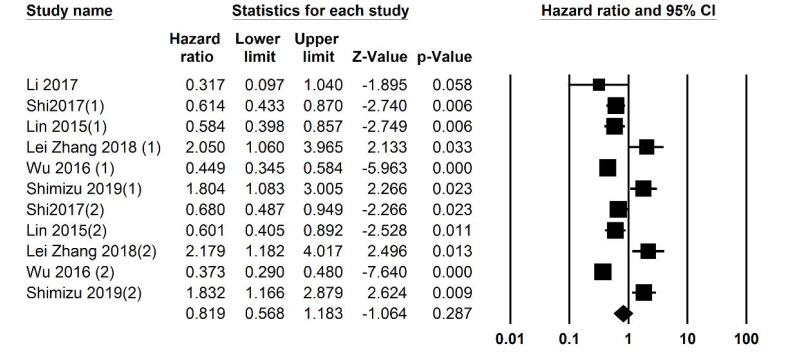
An overall analysis of the predictive value of lymphocyte to monocyte ratio in recurrences-free survival (RFS) analysis in studies which reporting RFS

**Supplementary Figure 3:**
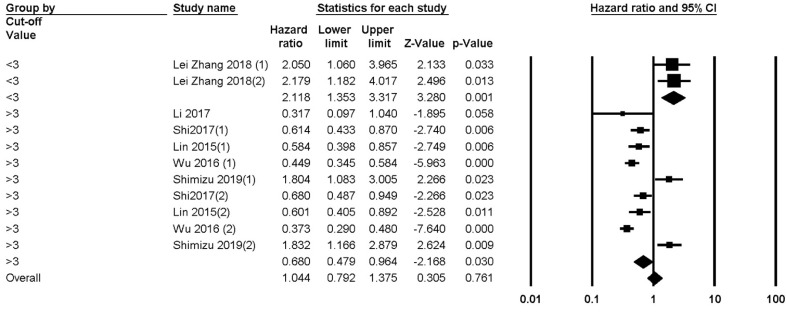
Subgroup analysis based on lymphocyte to monocyte ratio (LMR) cutoff s for the predictive value of LMR in recurrence free survival

**Supplementary Figure 4:**
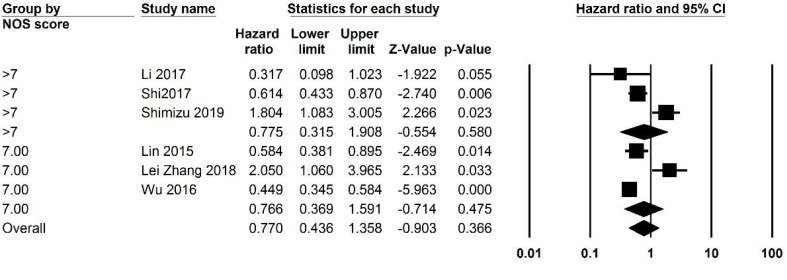
Subgroup analysis based on Newcastle-Ottawa Scale (NOS) regarding the predictive value of lymphocyte to monocyte ration in recurrence free survival

 Of the 12 included studies, 5 studies had reported RFS values and their relation to LMR. The heterogeneity of the studies was reported to be 88.926%; thus, subgroup analysis was performed. The number of patients being analyzed was 1472. The overall analysis showed a HR of 0.815 (95% CI 0.553-1.202, P=0.302, Figure-4). Subgroup analysis showed that studies with a cut-off of less than 3 HR was 2.118 (95% CI: 1.353-3.317, P=0.001). Studies with a cut-off value of more than 3 had an HR of 0.680 (95% CI, 0.964 - 0.479, P=0.03) (Figure-3B, S3, and S4).

####  1. 3.Disease -free Survival 

**Figure 5:**
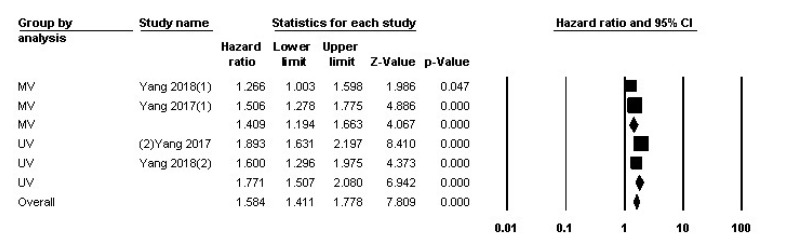
Overall and subgroup analysis based on multivariate and univariate analyses of the predictive value of lymphocyte to monocyte ratio (LMR) in disease-free survival

 Of the 12 studies included in this review, 2 studies had examined the relationship between LMR and DFS. Heterogeneity analysis revealed an I2 equal to 67.690 for fixed-effect analysis. The number of patients being analyzed was 1672. Figure-5 summarizes the HR of the studies and their univariate and multivariate analyses. It is shown that the overall analysis shows that the HR is 1.584 (95% CI: 1.411-1.778, P≤0.001). The funnel plot of the study is included in Figure-3C.

## Discussion

**Figure 6:**
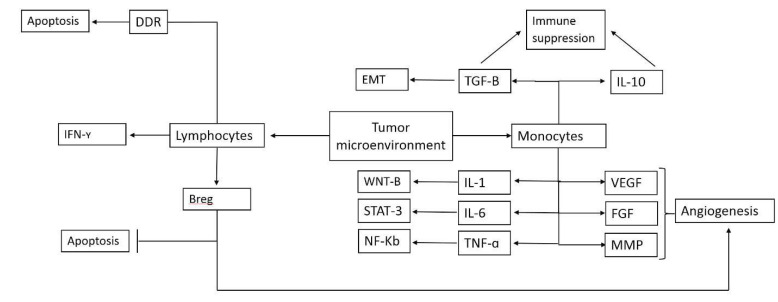
Relationship between tumor-infiltrating lymphocytes and tumor associated macrophages

In this systemic review, the significance of LMR regarding the prognosis of HCC was studied. LMR is regarded as a reliable surrogate marker of inflammatory responses and has previously been shown to be related to clinical outcomes over a wide range of conditions, ranging from trauma to malignant tumors and auto-immune diseases [24]. LMR elaborates on two important factors effective in tumor progression. The first is the immune response towards the tumor, which is shown by the number of lymphocytes, including Tumor-infiltrating lymphocytes [25]. These lymphocytes induce DNA damage response signaling in neoplastic cells, ultimately leading to endpoints such as apoptosis or excessive autophagy, which causes cell death [26]. On the contrary, monocytes associated with malignant tissue, or commonly termed Tumor-associated macrophages (TAM) are important drivers of cancer progression. Multiple in vitro studies have found that these cells contribute to angiogenesis and lymphangiogenesis, which results in increased tumoral cell proliferation, increased flux of intravascular fluid and ominously, and increased rates of distant metastasis [27]. Importantly, TAMs counter-act the immune system and have immunosuppressive functions [28]. A summary of the bio-cellular significance of TAMs and TILs is included in Figure-6. Tumor-infiltrating lymphocytes activate DNA damage response signaling and cause apoptosis [29]. Furthermore these interties secret IFN-Gamma which exerts direct anti-cancer effects [30]. Studies have also shown that some of these lymphocytes have pro-cancer effects, best shown by regulatory B lymphocytes that induce angiogenesis and inhibit apoptosis [31]. Tumor-associated macrophages are known cancer-promoting cells which secret mediators such as VEGF, FGF and MMP which promote angiogenesis, and TGF-beta that promotes EMT with a combined effect in increasing the rate of distant metastasis [32]. These cells secret IL-1, which increases proliferation in cancer cells [33]. TNF-alpha released by these cells results in the activation of the NF-Kb signaling pathway, which acts as a master regulator of inflammation [34]. IL-6 released by these cells leads to the activation of STAT3, causing the release of IL-10, which has proven to have immune suppression effects [35,36]. The importance of LMR is more obvious, where conventional grading and staging techniques are not adequate to predict OS, and also in instances which conventional tumor markers and studies are not associated with clinical outcome, and are rather associated with histopathological characteristics of tumors [37]. Original articles, systemic reviews, and meta-analysis focusing on a wide range of cancers including epithelial cancers [38], urological cancers [39], ovarian cancers [40], gastrointestinal cancers [41], and last but not least, hepatobiliary cancers [42], have found that LMR could be associated with OS, DFS, RFS and post-surgery life span, post-radiotherapy life span and quality of life. HCC is one of the deadliest cancers and much effort has been invested in trying to classify patients to good and poor outcome subgroups. Until now, studies focusing on molecular markers have generally found little success in identifying suitable markers [37]. One exception is alpha-fetoprotein which has been shown to be significantly associated with treatment outcome in patients undergoing a combination therapy with Sorafenib and chemoembolization [43], and is also a predictor of HCC recurrence following liver transplantation [44]. This marker is not without limitations; one important pitfall being the subgroup of HCCs with low-normal alpha-fetoprotein levels [45]. Accordingly, there has been extensive research on LMR in hepatocellular cancers, with novel studies being published rapidly. More so, couple of reviews have also focused on this subject matter. Song *et al*. performed a meta-analysis on 7 studies published between 2014 and 2017. The studies included in this systemic review had a combined patient number of 1718 [HR=0.31, 95%CI: 0.20–0.47], and LMR was associated with the length of OS and DFS/RFS [39]. This study had included 7 studies which were all performed in China, and had a significant heterogeneity (I2=74%) [39]. This study only reported data from China in languages other than English, and were not included in our study. We did not find any significant relation between LMR and OS, and only subgroup analysis yielded significant results for DFS and RFS. These differences in results could be due to the fact that our study included studies from other countries, such as Japan and France, and we included a large number of studies, including the analysis done regarding OS. In general, the number of studies in both is rather limited and is not enough to rule out or suggest the routine use of LMR as a prognostic marker, but both studies suggest that LMR may be beneficial for predicting DFS and RFS in patients suffering from HCC. Importantly, previous studies on the significance of LMR and other ratios of blood parameters have proven to be valuable prognostic markers for other cancers [39]; therefore, future studies using larger sample sizes and studies from different geographic locations, as well as multi-center studies can direct us towards better decision making. Worthy of attention, no single biomedical marker is adequately beneficial in detecting the prognosis of HCC, because of this LMR shows potential for further investigation, as it has shown great clinical relevance in other cancers, including other forms of hepatobiliary malignancies [46]. Moreover, even specific cellular markers of hepatic cells have not been beneficial in detecting the prognosis of HCC [47]. It should be noted that many other blood markers and parameters have been considered as prognostic indicators, including the platelet to neutrophil ratio, platelet to monocyte ratio, and the ratios of different leukocytes with various membrane identifiers to each other [48,49]. LMR is a considerable prognostic factor; however, little is known about the relative efficacy of LMR compared with other identifiers which may be an interest-bearing asset for the future. Our systematic review and meta-analysis had some limitations. The included studies in the RFS analysis were limited, which makes concern about the analysis accuracy of RFS. High heterogeneity of the variables may reduce the credibility of the results; however, a subgroup analysis was performed.

## Conclusion

 In this systemic review and meta-analysis, the prognostic value of the LMR in HCC was studied. There was no significant relationship between LMR and OS of the HCC, while subgroup analysis yielded significant results. DFS and RFS were correlated with LMR, although number of studies were limited compared with OS. Further studies could fruitfully explore the prognostic significance of the LMR in HCC.

## Acknowledgment

 The authors received no financial support for the research, authorship, and/or publication of this article.

## Conflict of Interest

 The authors declare that they have no conflict of interest.

**Table 1:** Characteristics of the Included Studies Which Have Reported the Relation of Lymphocyte to Monocyte Ratio

First author/Year	Study design	Regions	Enrollment period	Study population	No. Pts	Cut-off Value	Survival analysis	Analysis	NOS score	treatment
Lin [12]/ 2015	Retrospective	China	2002-2010	HBV-associated HCC patients	210	3.23	OS/ RFS	MV	7	Curative resection
Wu [13]/ 2016	Retrospective	China	January 2008-June 2013	HBV-associated HCC	450	3.77	OS/RFS	MV	7	Curative resection
Li [14]/ 2017	Retrospective	China	July 2008-July 2014	HBV-associated HCC	253	3 for OS	OS/RFS	UV/MV	8	Curative resection
						3.2 for RFS				
Hong [15]/ 2017	Retrospective	China	September 2008-June 2010	HBV-associated with advanced HCC	174	4.52	OS	UV/MV	7	Antiviral therapy with oral nucleos(t)ide
Shi [16]/ 2017	Retrospective	China	2008-2011	Patients with HCC	271	4.5	OS/TTR	UV/MV	8	Curative resection
Chen [17]/ 2017	N/A	China	September 2008-June 2010	Advanced HBV- associated HCC	174	4.52	OS	UV/MV	N/A	N/A
Yang [18]/ 2017	Retrospective	China	January 2004-June 2011	HBV- and HCV-associated HCC	1020	3.23	DFS	UV/MV	6	Curative resection
Conroy [19]/ 2018	Retrospective	France	October2007-September 2015	Advanced HCC patients	161	3	OS	UV/MV	6	Prior to treatment with Sorafenib
Yang [20]/ 2018	Retrospective	China	April 2004-April 2012	HCC patients	652	4.01	DFS/OS	UV/MV	7	Curative resection
Takagi [21]/ 2018	N/A	Japan	N/A	HCC patients with low CLIP scores	329	4.35	Overall survival	UV/MV	N/A	Curative resection
Zhang [22]/ 2019	Prospective	China	January 2011-December 2013	HCC patients	230	2	RFS/OS	UV/MV (not having MV for OS)	7	Hepatectomy and adjuvant chemotherapy
Shimizu [23]/ 2019	Retrospective	Japan	April 2005-December 2015	HCC patients with a low CLIP score	329	4.35	RFS/OS	MV/UV	8	Curative Resection

**CLIP: **Cancer of the Liver Italian Program; **DFS: **Diseases Free Survival; **HCC: **Hepatocellular Carcinoma; **HBV: **Hepatitis B Virus; **HCV: **Hepatitis C Virus; **MV: **Multivariable; **N/A: **Not Available; **OS:** Overall Survival; **UV:** Univariable; **RFS: **Recurrence Free Survival; **TTR:** Time to Recurrence

**EMT: **Epithelial Mesenchymal Transformation; ** DDR:** DNA Damage Response; **IFN: **Interferon; **TGF-B: **Transforming Growth Factor Beta; **TNF-alpha:** Tumor Necrosis Factor Alpha; **STAT3:** Signal Transducer and Activator of Transcription 3; **NF-kb: **Nuclear Factor Kappa-light-chain-enhancer of Activated B Cells; **MMP: **Matrix Metallopeptidases; **FGF:** Fibroblast Growth Factor

## References

[1] Ghouri YA, Mian I, Rowe JH (2017). Review of hepatocellular carcinoma: Epidemiology, etiology, and carcinogenesis. J Carcinog.

[2] Yu LX, Ling Y, Wang HY (2018). Role of nonresolving inflammation in hepatocellular carcinoma development and progression. NPJ Precis Oncol.

[3] Volcic M, Karl S, Baumann B, Salles D, Daniel P, Fulda S (2012). NF-κB regulates DNA double-strand break repair in conjunction with BRCA1-CtIP complexes. Nucleic Acids Res.

[4] Taniguchi K, Karin M (2018). NF-kappaB, inflammation, immunity and cancer: coming of age. Nat Rev Immunol.

[5] Adam JK, Odhav B, Bhoola KD (2003). Immune responses in cancer. Pharmacol Ther.

[6] Chen G, Zhu L, Yang Y, Long Y, Li X, Wang Y (2018). Prognostic Role of Neutrophil to Lymphocyte Ratio in Ovarian Cancer: A Meta-Analysis. Technol Cancer Res Treat.

[7] Zhao J, Huang W, Wu Y, Luo Y, Wu B, Cheng J (2020). Prognostic role of pretreatment blood lymphocyte count in patients with solid tumors: a systematic review and meta-analysis. Cancer cell international.

[8] Liu L, Gong Y, Zhang Q, Cai P, Feng L (2019). Prognostic Roles of Blood Inflammatory Markers in Hepatocellular Carcinoma Patients Taking Sorafenib A Systematic Review and Meta-Analysis. Front Oncol.

[9] Mao Y, Chen D, Duan S, Zhao Y, Wu C, Zhu F (2018). Prognostic impact of pretreatment lymphocyte-to-monocyte ratio in advanced epithelial cancers: a meta-analysis. Cancer Cell Int.

[10] Li J, Cheng Y, Ji Z (2019). Prognostic value of pretreatment lymphocyte-to-monocyte ratio in patients with urologic tumors: A PRISMA-compliant meta-analysis. Medicine.

[11] Wells G. The Newcastle-Ottawa Scale (NOS) for assessing the quality of nonrandomised studies in meta-analysis. http://wwwohrica/programs/clinical_epidemiologyoxfordhtm. 2004.

[12] Djordjevic D, Rondovic G, Surbatovic M, Stanojevic I, Udovicic I, Andjelic T (2018). Neutrophil-to-Lymphocyte Ratio, Monocyte-to-Lymphocyte Ratio, Platelet-to-Lymphocyte Ratio, and Mean Platelet Volume-to-Platelet Count Ratio as Biomarkers in Critically Ill and Injured Patients: Which Ratio to Choose to Predict Outcome and Nature of Bacteremia?. Mediators Inflamm.

[13] Badalamenti G, Fanale D, Incorvaia L, Barraco N, Listi A, Maragliano R (2019). Role of tumor-infiltrating lymphocytes in patients with solid tumors: Can a drop dig a stone?. Cell Immunol.

[14] Green AR, Aleskandarany MA, Ali R, Hodgson EG, Atabani S, De Souza K (2017). Clinical Impact of Tumor DNA Repair Expression and T-cell Infiltration in Breast Cancers. Cancer Immunol Res.

[15] Riabov V, Gudima A, Wang N, Mickley A, Orekhov A, Kzhyshkowska J (2014). Role of tumor associated macrophages in tumor angiogenesis and lymphangiogenesis. Frontiers in physiology.

[16] Aras S, Zaidi MR (2017). TAMeless traitors: macrophages in cancer progression and metastasis. Br J Cancer.

[17] Goto W, Kashiwagi S, Asano Y, Takada K, Takahashi K, Hatano T (2018). Predictive value of lymphocyte-to-monocyte ratio in the preoperative setting for progression of patients with breast cancer. BMC Cancer.

[18] Kano S, Homma A, Hatakeyama H, Mizumachi T, Sakashita T, Kakizaki T (2017). Pretreatment lymphocyte-to-monocyte ratio as an independent prognostic factor for head and neck cancer. Head Neck.

[19] Song W, Tian C, Wang K, Zhang R-J, Zou S-B (2017). The pretreatment lymphocyte to monocyte ratio predicts clinical outcome for patients with hepatocellular carcinoma: A meta-analysis. Scientific reports.

[20] Gong J, Jiang H, Shu C, Hu M-Q, Huang Y, Liu Q (2019). Prognostic value of lymphocyte-to-monocyte ratio in ovarian cancer: a meta-analysis. Journal of ovarian research.

[21] Zhang J, Chen L, Zhou R, Sun H, Liao Y, Liao W (2016). Pretreatment lymphocyte monocyte ratio predicts long-term outcomes in patients with digestive system tumor: a meta-analysis. Gastroenterol Res Pract.

[22] Zhang Y, Shi S-M, Yang H, Yang L-X, Wang Z, Li X-D (2019). Systemic inflammation score predicts survival in patients with intrahepatic cholangiocarcinoma undergoing curative resection. J Cancer.

[23] Liu L, Zhao Y, Jia J, Chen H, Bai W, Yang M (2016). The prognostic value of alpha-fetoprotein response for advanced-stage hepatocellular carcinoma treated with sorafenib combined with transarterial chemoembolization. Sci Rep.

[24] Fatourou EM, Suddle AR, Heneghan MA (2018). Alpha-fetoprotein as a predictor of hepatocellular carcinoma recurrence following liver transplantation. Hepatoma Res.

[25] Carr BI, Pancoska P, Branch RA (2010). Low alpha-fetoprotein hepatocellular carcinoma. J Gastroenterol Hepatol.

[26] Peng D, Lu J, Hu H, Li B, Ye X, Cheng N (2019). Lymphocyte to Monocyte Ratio Predicts Resectability and Early Recurrence of Bismuth-Corlette Type IV Hilar Cholangiocarcinoma. J Gastroint Surg.

[27] Zhao Y-J, Ju Q, Li G-C (2013). Tumor markers for hepatocellular carcinoma. Mol Clin Oncol.

[28] Passacquale G, Vamadevan P, Pereira L, Hamid C, Corrigall V, Ferro A (2011). Monocyte-platelet interaction induces a pro-inflammatory phenotype in circulating monocytes. PloS one.

[29] Lisman T (2018). Platelet-neutrophil interactions as drivers of inflammatory and thrombotic disease. Cell Tissue Res.

[30] Lin Z-X, Ruan D-Y, Li Y, Wu D-H, Ma X-K, Chen J (2015). Lymphocyte-to-monocyte ratio predicts survival of patients with hepatocellular carcinoma after curative resection. World J Gastroenterol.

[31] Wu SJ, Lin YX, Ye H, Li FY, Xiong XZ, Cheng NS (2016). Lymphocyte to monocyte ratio and prognostic nutritional index predict survival outcomes of hepatitis B virus-associated hepatocellular carcinoma patients after curative hepatectomy. J Surg Oncol.

[32] Li G-J, Ji J-J, Yang F, Xu H-W, Bai Y (2017). Preoperative lymphocyte-to-monocyte ratio predicts survival in primary hepatitis B virus-positive hepatocellular carcinoma after curative resection. Onco Targets Ther.

[33] Hong YF, Chen ZH, Wei L, Ma XK, Li X, Wen JY (2017). Identification of the prognostic value of lymphocyte-to-monocyte ratio in patients with HBV-associated advanced hepatocellular carcinoma. Oncol Lett.

[34] Shi S, Chen Q, Ye L, Yin D, Li X, Dai Z (2017). Prognostic value of systemic inflammation score in patients with hepatocellular carcinoma after hepatectomy. Oncotarget.

[35] Chen Z-H, Hong Y, Ma X-k, Li X, Wu D-h, Lin Q, et al. Identification of prognostic value of lymphocyte-to-monocyte ratio in patients with advanced HBV-associated hepatocellular carcinoma. Oncol Lett. 2016. 10.3892/ol.2017.6420 PMC5530031 28789436

[36] Yang T, Zhu J, Zhao L, Mai K, Ye J, Huang S (2017). Lymphocyte to monocyte ratio and neutrophil to lymphocyte ratio are superior inflammation-based predictors of recurrence in patients with hepatocellular carcinoma after hepatic resection. J Surg Oncol.

[37] Conroy G, Salleron J, Belle A, Bensenane M, Nani A, Ayav A (2017). The prognostic value of inflammation-based scores in advanced hepatocellular carcinoma patients prior to treatment with sorafenib. Oncotarget.

[38] Yang Y-T, Jiang J-H, Yang H-J, Wu Z-j, Xiao Z-M, Xiang B-D (2018). The lymphocyte-to-monocyte ratio is a superior predictor of overall survival compared to established biomarkers in HCC patients undergoing liver resection. Sci Rep.

[39] Takagi Y, Shimizu T, Ishizuka M, Shiraki T, Mori S, Iso Y (2018). Preoperative lymphocyte-to-monocyte ratio is useful for stratifying the prognosis of HCC patients with low CLIP scores. HPB.

[40] Zhang L, Chen QG, Li SQ, Zhang J, Min QH, Gao QF (2019). Preoperative fibrinogen to prealbumin ratio as a novel predictor for clinical outcome of hepatocellular carcinoma. Future Oncol.

[41] Shimizu T, Ishizuka M, Park KH, Shiraki T, Sakuraoka Y, Mori S (2019). Preoperative lymphocyte-to-monocyte ratio is useful for stratifying the prognosis of hepatocellular carcinoma patients with a low Cancer of the Liver Italian Program score undergoing curative resection. Ann Gastroenterol Surg.

[42] Gilmore E, McCabe N, Kennedy RD, Parkes EE (2019). DNA Repair Deficiency in Breast Cancer: Opportunities for Immunotherapy. J Oncol.

[43] Ni L, Lu J (2018). Interferon gamma in cancer immunotherapy. Cancer medicine.

[44] Shen M, Wang J, Ren X (2018). New Insights into Tumor-Infiltrating B Lymphocytes in Breast Cancer: Clinical Impacts and Regulatory Mechanisms. Front Immunol.

[45] Han Y, Guo W, Ren T, Huang Y, Wang S, Liu K (2019). Tumor-associated macrophages promote lung metastasis and induce epithelial-mesenchymal transition in osteosarcoma by activating the COX-2/STAT3 axis. Cancer Lett.

[46] Kondo M, Yamato M, Takagi R, Namiki H, Okano T (2013). The regulation of epithelial cell proliferation and growth by IL-1 receptor antagonist. Biomaterials.

[47] Mancino A, Lawrence T (2010). Nuclear factor-kappaB and tumor-associated macrophages. Clin Cancer Res.

[48] Hutchins AP, Diez D, Miranda-Saavedra D (2013). The IL-10/STAT3-mediated anti-inflammatory response: recent developments and future challenges. Brief Funct Genomics.

[49] Wan S, Zhao E, Kryczek I, Vatan L, Sadovskaya A, Ludema G (2014). Tumor-associated macrophages produce interleukin 6 and signal via STAT3 to promote expansion of human hepatocellular carcinoma stem cells. Gastroenterology.

